# Worker health in the historical-cultural clinic: a psychosocial discussion on Regulatory Standard-1

**DOI:** 10.47626/1679-4435-2025-1437

**Published:** 2025-08-25

**Authors:** Larissa Gomes Pereira

**Affiliations:** 1 Lev Histórico-cultural, Fortaleza, CE, Brazil

**Keywords:** occupational health, psychology, pathologic processes, saúde ocupacional, psicologia, processos patológicos

## Abstract

This opinion article examines the intersection between Worker Health and the Historical-Cultural Clinic, emphasizing the need to analyze and question the psychosocial reality of workers in alignment with Regulatory Standard-1 (NR-1). Highlighting the importance of collaboration among professionals working directly and indirectly in Worker Health, this discussion is framed within the Historical-Cultural Psychology approach. A questionnaire is presented as a tool to support the initial assessment of organizations in accordance with NR-1. This survey can help identify and enhance control over occupational risks in the workplace, ultimately ensuring the health and safety of workers. The article concludes by underscoring the need to expand professional engagement in this field.

## Abstract (pt)

Este artigo de opinião analisa a interface entre a saúde do trabalhador e a Clínica Histórico-Cultural, discutindo a importância de analisar e questionar a realidade psicossocial dos trabalhadores em consonância com a Norma Regulamentadora-1. Destacando a importância de um trabalho conjunto entre os profissionais que atuam direta e indiretamente com a saúde do trabalhador, a partir da Psicologia Histórico-Cultural, é apresentado um questionário para auxiliar na avaliação inicial sobre as organizações, de acordo com a Norma Regulamentadora-1. Tal levantamento pode identificar e promover um maior controle sobre os riscos ocupacionais nos ambientes de trabalho, com o objetivo de garantir a segurança e a saúde dos trabalhadores. Conclui-se, assim, que é fundamental expandir a atuação dos profissionais nesse campo.

## INTRODUCTION

Work has undergone constant transformations under neoliberal management, which, rooted in a meritocratic and individualistic bourgeois ideology, has contributed to the growing deterioration of the physical and mental health of the working class. Similarly Han^1^ argues that exploitation is no longer merely imposed by an external force demanding efficiency but has instead become internalized by the workers themselves. This scenario has led to a reduction in job opportunities, as multiple roles and tasks that were once distributed across various sectors are now assigned to a single individual. This intensification of labor exploitation further exacerbates occupational illnesses.

Mansano & Carvalho^2^ explain that new politics of subjectivation are emerging in the contemporary labor landscape. While disciplinary society and its control mechanisms have not been entirely overcome, their contours are rapidly dissolving and reorganizing in increasingly complex ways, now intertwined with forms of subjective control. This dynamic scenario, marked by both progress and new forms of confinement, has had a significant impact on the mental health of workers. The rise in psychopathologies such as depression, stress, and panic disorders reflects this shift, contributing to an increase in work-related absences due to health issues.^2^ These conditions are closely linked to pathological processes that disrupt the normal physiological state of the human body.

Furthermore, the intrusion of work into private life has become even more prevalent, especially after the COVID-19 pandemic.^3^ As Lucca & Magalhães point out,^4^ managerialism, driven by the pursuit of short-term results, has intensified workplace tensions and individualized labor relations, leading to increased suffering, as evidenced by the sharp rise in mental health disorders. Notably, the surge in psychological distress has paralleled changes in the labor market, particularly in the most socially undervalued sectors and those most affected by the rise of gig work.^5^

Organizational Health has linked the rise in cases to poor management of Psychosocial Risk Factors at Work (PRFW) by organizations.^4^ In this context, the Risk Management Program (RMP), mandated by Regulatory Standard-1 (NR-1) of the Brazilian Ministry of Labor, aims to identify, assess, and control occupational risks in the workplace, ensuring the health and safety of workers.

However, psychosocial factors in the workplace must be examined in relation to work organization, including aspects such as job content, work pace, workload, degree of autonomy, communication and participation in decision-making, conflicts, and career development.^4^ Therefore, these factors should be analyzed by occupational health professionals,^4^ and psychology is one of the scientific fields specializing in this area.

In this context, an analysis of NR-l from the perspective of Historical-Cultural Psychology (HCP) aims to identify, assess, and control occupational risks in the workplace, ensuring the health and safety of workers. As Lucca & Magalhães argue,^4^ although NR-l does not provide a detailed approach to psychosocial factors, diagnosing and managing these factors is essential for creating a healthier work environment.

### HCP IN CLINICAL PRACTICE: ADDRESSING THE PSYCHOLOGICAL DISTRESS OF WORKERS

According to Accarini,^6^ data from the Brazilian Ministry of Social Security indicate that nearly one-third (30.67%) of work-related absences and disability benefits are due to mental disorders caused by workplace conditions. Workers who do not receive support from Psychosocial Care Centers (CAPS) are often referred to private health plans or independent mental health professionals, such as psychologists and psychiatrists, in search of psychological and pharmacological treatment.

Psychologists often see patients with diagnoses or suspected diagnoses of disorders such as burnout, depression, and anxiety that are directly related to the work environment. The alarming rate of worker illness, often a result of excessive workload, constant pressure, and lack of recognition, highlights the psychosocial impact of work on mental health.

As Franco et al.^7^ have already pointed out, the loss of the social purpose of work leads to a depletion of its meaning for those who perform it, with negative effects on mental health due to the contradictions between modernization and the increasing precarization of work and social conditions. As Mészáros^8^ argues, the structural crisis of capitalism has permeated all aspects of society, but no domain has been as stripped of meaning as work itself. From the perspective of HCP, there is no doubt that the historical expansion of capital has reduced the lives of workers to mere labor power.

Moreover, as Marques et al.^9^ explain, work processes function as mechanisms of capitalist control, facilitating the extraction of surplus value without regard for the health of those who generate wealth through their labor. To develop a dialectical and structural understanding of occupational risk factors, it is essential to analyze the various manifestations of worker fatigue within their unique lived experiences. This perspective forms the foundation of the current reflections produced by the Historical-Cultural Clinic (HCC) in its care for the ailing working class.

In addressing the health of workers and truly acknowledging the suffering experienced by patients, HCC therapists explore the origins of psychological illness^10^ in order to guide individuals through a therapeutic process. As Melo^11^ states, “we introduce mediators and build new possibilities, walking alongside the individual in their journey,” with the goal of offering, through HCC, a way to overcome the crisis that led to their psychological distress (pathological process).

### UNDERSTANDING THE ROOTS OF WORK-RELATED ILLNESS IN THE STRUCTURE THAT PRODUCES IT

Marx^12^ has already analyzed how the structure of the capitalist system keeps workers in harmful working conditions. In the same vein, a large-scale study published in 1975 had already demonstrated the links between psychological stress in the workplace, its impact on emotions, physiological health, and the illnesses reported by workers.^13^ The study confirmed that certain factors — such as high workload, low autonomy, and lack of social support — can intensify these stress responses. When prolonged, these tensions not only undermine psychological well-being but can also trigger physical health problems, including cardiovascular diseases and mental health disorders.^13^

Caplan et al.^13^ analyzed 23 different professions and surveyed 2,010 workers to advance the development of an empirically valid theory on psychological stress and its consequences. Their hypothesis — that occupational stressors generate emotional strain — was strongly confirmed. Data showed negative emotional responses to workplace stress manifest as anxiety, irritation, frustration, and demotivation. The authors highlight the need to identify and mitigate these stressors to foster a healthier and more productive work environment.

Moreover, workers become ill within material circumstances shaped by the historical period in which they live. In Marxist theory, the working class consists of individuals who have no choice but to sell their labor, while the middle class includes small business owners and independent professionals.^14^ However, with increasing labor precarity, many salaried middle-class workers are being pushed closer to the condition of the working class due to the uncontrollable nature of capitalist socio-metabolism.^15^ In this sense, anyone dependent on selling their labor is subject to market conditions.

Thus, rising worker illness rates are a direct result of unrestrained exploitation,^16^ with suicide^12^ emerging as an extreme response to the inability to endure an increasingly dehumanizing social structure. After all, no body can withstand relentless labor without breaking down, and class society shows that illness is an inevitable path for the proletariat. As Wright^17^ states, “the oppressed possess the power that comes from the human capacity for physical resistance,” but continuous resistance takes its toll, ultimately leading to illness.

This Marxist perspective on the social division of labor was also indirectly confirmed by Caplan et al.^13^, who highlighted significant differences in the occupational health impacts on middle-class and working-class employees regarding stress and strain. Several interrelated stressors — such as skill underutilization, low participation, low job complexity, poor alignment between complexity and responsibility for people, and role ambiguity — were particularly prevalent among assembly line workers, forklift operators, and machine operators.

In contrast, these stressors were significantly lower among teachers, family physicians, and other professionals. The study also found that the most satisfying occupations included university professors, family physicians, and administrative supervisors, while scientists exhibited the lowest blood pressure levels among the eight occupations measured. Overall, assembly workers and employees in mechanized production lines experienced the highest levels of stress and strain compared to any other occupation analyzed.^13^

### PSYCHOSOCIAL WORK FACTORS FROM THE PERSPECTIVE OF HCP

From the perspective of HCP, work is not merely a productive activity but also a mediating process in human development.^18^ Therefore, to understand psychosocial work factors, HCP examines the interaction between the individual, culture, and historical-material conditions, as these elements form the foundation for the construction of subjectivity and Higher Psychological Functions (HPF). According to Souza & Andrada,^19^ HPF transition from natural to cultural when mediated, as individuals, through social integration, transform the social development they experience into psychological functions that become intrinsic to their personality.

Memory, consciousness, thought, will, and emotion are examples of HPF that interact within a network of relationships, forming a dynamic system.^19^ These connections shape the construction of new meanings and interpretations throughout development, driving qualitative transformations in the individual. Thus, it is assumed that an individual’s activity within capitalist sociability is influenced by psychosocial work factors, which must be understood through the complex interplay of social, material, and cultural conditions specific to a given historical period.

Building on this theoretical framework, the psychosocial work factors highlighted by Lucca & Magalhães,^4^ when analyzed from the perspective of HCP, can be explored through questions such as 1. Does the nature of the work allow for active, creative, and conscious participation, fostering worker development? 2. Is the workload exhausting, affecting higher psychological functions and the individual’s physical integrity? 3. Can the worker actively participate in decisions regarding their role? Does communication allow for questioning and adjustments, or is it limited to rigid task distribution? 4. Are personal issues, including health and family-related concerns, acknowledged by the company, or are they ignored? 5. Does the salary provide the employee with a dignified life, ensuring access to health care, food, transportation, and other essential needs?

These questions can serve as an initial assessment of whether organizations comply with NR-1 and can be incorporated into a questionnaire useful for professionals evaluating workplace conditions. This survey can help identify and conduct a qualitative assessment of occupational risks related to the health of workers. In agreement with Lucca & Magalhães,^4^ although NR-1 does not explicitly address psychosocial factors, it is essential to develop methods for diagnosing and assessing these factors to create a work environment that does not contribute to illness.

### WORKER HEALTH NEEDS TO BE A MORE WIDESPREAD CONCERN

Through an analysis of the RMP, a requirement of NR-1, this study sought to explore a potential approach for investigating occupational risks in the workplace to ensure the health and safety of workers. However, from a dialectical perspective, the limitations of NR-1 within a social context shaped by worker exploitation also became evident, as any form of wage labor inherently involves some degree of surplus value extraction.^14^ Based on this premise, analyzing workplace environments highlights the undeniable need for change in the world of work to prevent illness and psychological distress among workers. Historically, such efforts have led to greater security and oversight of human rights^20^ in the labor sphere in some countries, yet they have not eradicated the exploitation and dehumanization inherent to capital accumulation.

This article serves as a call to action. As Arruzza et al.^21^ emphasize, political struggle must encompass all issues in pursuit of a society that does not defend the interests of a few at the expense of exploiting others’ labor. Nonetheless, within the current social structure, the creation of laws and regulations to manage and promote dignified working conditions remains crucial. NR-1 represents progress in this regard, but it does not address the root problem: worker exploitation and the resulting vulnerability to illness due to precarious psychosocial conditions shaped by the social division of labor.

Professionals working in Worker Health must push the discussion forward: our current efforts only address part of the problem. This limitation arises from the very structure of capitalism,^15^ which traps us in the fatalistic notion that there is only so much we can do. However, rather than accepting these constraints, both CHC psychologists and professionals in other fields directly or indirectly involved in Organizational Health must critically reflect on how to collectively build a counterpoint to the capitalist system — one that not only generates psychopathological processes in our patients but also affects us as members of the working class.
